# Lactic acidosis associated with metformin in patients with moderate to severe chronic kidney disease: study protocol for a multicenter population-based case-control study using health databases

**DOI:** 10.1186/s12882-019-1389-8

**Published:** 2019-05-30

**Authors:** Consuelo Pedrós, Mónica Ávila, Ainhoa Gómez-Lumbreras, Marcela Manríquez, Rosa Morros, ALIMAR-C2 Study Group, Consuelo Pedrós, Mónica Ávila, Marcela Manríquez, Sebastián Videla, Rosa Morros, Ainhoa Gómez-Lumbreras, Oriol Prat-Vallverdú, Inmaculada Fuentes, Angélica Valderrama, Cristina Aguilera, Ana María Barriocanal, Joaquín Sáez-Peñataro, Rosa Antonijoan, Claudia E. Delgado, Lucía Llanos, Leonor Laredo, Mónica Aguilar, Teresa Sanz, Montserrat Hernández, José C. Estévez, Sergio Ruiz, Lluís Murgui, Xavier Corbella, Xavier Fulladosa, Manuel Pérez-Maraver, Virginia Alonso, Manel Mata-Cases

**Affiliations:** 1 Clinical Pharmacology Department, Bellvitge University Hospital, L’Hospitalet de Llobregat, Barcelona, Spain; 2 Bellvitge Biomedical Research Institute (IDIBELL), L’Hospitalet de Llobregat, Barcelona, Spain; 3 Clinical Research and Clinical Trials Unit, Bellvitge Biomedical Research Institute (IDIBELL), L’Hospitalet de Llobregat, Barcelona, Spain; 4 Medicines Research Unit, Foundation University Institute for Primary Health Care Research Jordi Gol i Gurina (IDIAPJGol), Barcelona, Spain; 5 Department of Medical Sciences, Girona University, Girona, Spain; 6 Departament de Farmacologia, Terapèutica i Toxicologia, Universitat Autònoma de Barcelona, Barcelona, Spain; 7 Catalan Health Institute, Barcelona, Spain

**Keywords:** Metformin, Type 2 diabetes mellitus, Chronic kidney failure, Lactic acidosis, Case-control studies, Electronic health records

## Abstract

**Background:**

The use of metformin in patients with type 2 diabetes mellitus has been associated with lactic acidosis. However, the information available in patients with moderate-severe chronic kidney disease is scarce.

**Methods:**

The ALIMAR-C2 study is a case-control study to assess the association between metformin and lactic acidosis in patients with type 2 diabetes mellitus and moderate-severe chronic kidney disease. The study will be performed with computerized registered electronic health records from eight Spanish hospitals linked to their corresponding primary care health areas from 2010 to 2016, comprising approximately 22.1 million person-years of follow-up. Logistic regression will be used to assess the crude and adjusted risk of lactic acidosis associated with metformin use overall and stratifying by use and dose categories, and chronic kidney disease stage. The overall case fatality rate of lactic acidosis, as well as the case fatality rate stratified by chronic kidney disease stage, will be calculated.

**Discussion:**

The ALIMAR-C2 study will provide useful information about the risk of lactic acidosis in type 2 diabetes mellitus patients with renal impairment using metformin.

**Electronic supplementary material:**

The online version of this article (10.1186/s12882-019-1389-8) contains supplementary material, which is available to authorized users.

## Background

Metformin is the first-line treatment for type 2 diabetes mellitus (DM2) when diet and exercise do not result in adequate glycaemia control, especially in overweight patients [1]. It was the first antidiabetic agent that showed to reduce diabetic complications and overall mortality in overweight and obese DM2 patients [2].

Its use has been related to the occurrence of lactic acidosis (LA), a rare but severe adverse effect, especially in patients with renal disease [3]. For this reason, it is contraindicated in patients with an estimated glomerular filtration rate (eGFR) below 30 mL/min.

Nevertheless, the association between metformin and LA has been a controversial issue due to conflicting results from different studies. The recommendation for patients with eGFR between 30 and 60 mL/min has been a matter of debate [4]. Currently, some studies analyzing the risk of LA failed to show an increase of risk with metformin use [5–7].

Information regarding patients with impaired renal function is scarce. In this line, some observational studies have shown an increase of incidence of LA in patients exposed to metformin in parallel to the degree of impairment of renal function [8], as well as an increase of LA risk in patients with eGFR < 60 mL/min mainly due to higher risk in patients with eGFR < 45 mL/min [9]. More recently, a study commissioned by the European Medicines Agency (EMA) to assess the use and safety of metformin in real clinical practice in patients with or without renal failure showed a greater risk of LA in metformin users than in other glucose-lowering agent users. Additionally, the incidence rates of LA increased with decreasing baseline eGFR [10]. Afterward, the EMA carried out a referral procedure in order to review the evidence justifying the contraindication of metformin use in chronic kidney disease (CKD). In October 2016, that safety review concluded that metformin could be used in patients with moderately reduced kidney function (eGFR 30–59 mL/min) [11].

Before the beginning of European referral procedure, the ALIMAR-C2 study (“*Riesgo de Acidosis Láctica asociada al uso de MetforminA en pacientes diabéticos tipo 2 con enfermedad Renal crónica moderada-severa: estudio de Casos y Controles*”) was designed to provide further data on risk of LA associated with metformin use in diabetic patients with CKD.

The aim of this publication is to present the study protocol in detail.

## Methods/design

### Aims

The primary objective of the ALIMAR-C2 study is to assess the association between the use of metformin and LA in patients with DM2 and moderate to severe CKD.

Secondary objectives include: (1) to evaluate the association according to daily dose of metformin and stage of CKD, to analyse the effect of comorbidities and concomitant medications, and to estimate the case fatality rate and the incidence of admission to critical care units of LA; (2) to assess the association of LA with the use of other non-insulin antidiabetic drugs (NIADDs) and insulin; and (3) to analyse the existence of a detection bias that affects the diagnosis of LA depending on the exposure to metformin.

### Study design

This is a population-based case-control study using hospital healthcare databases linked to primary health care databases.

### Setting

The study will be conducted through collaboration of researchers at eight hospitals from Madrid (Ramón y Cajal University Hospital, Fundación Jiménez Díaz University Hospital, and Hospital Clínico San Carlos) and Catalonia (Bellvitge University Hospital [coordinating centre], Hospital Germans Trias i Pujol, Hospital Clinic of Barcelona, University Hospital Vall d’Hebron, and Hospital de la Santa Creu i Sant Pau) and their corresponding public institutions for primary care (Institut Universitari d’Investigació en Atenció Primària [IDIAP] Jordi Gol in Catalonia, and Gerencia Asistencial de Atención Primaria in Madrid). Initially, other eight hospitals were invited to participate in the study but they were excluded after a feasibility assessment. Table 1 describes the population covered, the study period, and the population-time of follow-up in each participant centre. Overall, the study includes approximately 22.1 million person-years of follow-up.

**Table 1:** Overall description of data sources and study period

Database/Hospital			Catalonia						Madrid			
			Bellvitge University Hospital	Hospital Germans Trias i Pujol	Hospital Clinic of Barcelona	University Hospital Vall d’Hebron	Hospital de la Santa Creu i Sant Pau	SIDIAP	Hospital Clínico San Carlos	Ramón y Cajal University Hospital	Fundación Jiménez Díaz University Hospital	Gerencia de Atención Primaria
Population covered (inhabitants)			373.000	810.000	540.000	450.000	403.000	N/A	366.000	574.000	424.000	N/A
Database type (Electronic medical register)	Hospital	Data available	+	+	+	+	+	N/A	+	+	+	N/A
		Clinical data	DW	Hospital DW	Hospital DW	Hospital DW	Hospital DW	N/A	Hospital DW	CMBD	Hospital DW	N/A
		Laboratory data	DW	Hospital DW	ServoLab	*Modulab, clinical records*	*Openlab*	N/A	Laboratory database	Openlab	Hospital DW	N/A
	Primary care	Data available	+	N/A	N/A	N/A	N/A	+	N/A	N/A	N/A	+
		Clinical data	DW	N/A	N/A	N/A	N/A	ECAP	N/A	N/A	N/A	Ap-Madrid
		Laboratory data	DW	N/A	N/A	N/A	N/A	ECAP	N/A	N/A	N/A	Ap-Madrid
Starting year of electronic data collection			2010	2008	2003	2009	2011	2006	2009	2009	2009	2002
Study period			April 2011–December 2016	January 2014–December 2016	January 2010–December 2016	January 2010–December 2016	January 2012–December 2016	January 2010–December 2016	January 2010–December 2015	January 2010–December 2015	January 2010–December 2016	January 2010–December 2016
Disease and procedure coding system			ICD 9	ICD 9	ICD 9	ICD 9	ICD 9	ICD 10	ICD 9	ICD 9	ICD 9	ICPC-2
Hospital admission and discharge data available			Yes	Yes	Yes	Yes	Yes	No	Yes	Yes	Yes	No
Drug dictionary code			WHO-ATC, National Drug Codes	N/A	N/A	N/A	N/A	WHO-ATC, National Drug Codes	N/A	N/A	N/A	WHO-ATC, National Drug Codes
Drug information			Prescriptions, data on strength and length	N/A	N/A	N/A	N/A	Prescriptions, data on strength and length	N/A	N/A	N/A	Prescriptions, data on strength and length

*DW* Data warehouse, *CMBD*, *Conjunto mínimo básico de datos* (Basic minimum set of data), *ECAP*, *Estació clínica d’atenció primària* (electronic health records in primary healthcare), *Ap-Madrid* Electronic Medical Record of Primary Care of Madrid, *ICD 9* International Classification Diseases, 9th revision, *ICD 10* International Classification Diseases, 10th revision, *ICPC-2* International Classification Diseases, 2th revision, *N/A* not applicable

### Data sources

The Catalonian hospitals have data warehouses (DWs) with administrative, clinical and laboratory data collected during clinical practice. Systems Applications and Products in Data Processing Business Objects (SAP BO) is used for the data mining of the DWs. Hospital Clinic of Barcelona, Hospital de la Santa Creu i Sant Pau, and University Hospital Vall d’Hebron do not have some laboratory results fully integrated with their corresponding DWs.

DW of Bellvitge University Hospital is the only one that also integrates the information corresponding to clinical and primary care data. For the other Catalan hospitals, the data source for primary care in Catalonia is SIDIAP (Information System for Research in Primary Care) which contains anonymized clinical information of all the primary care centres of the Institut Català de la Salut (ICS) [12]. It covers more than 5.8 million patients (approximately 80% of the Catalan population, which represents more than 10% of the Spanish population). The information comes from *Estació clínica d’atenció primària* (ECAP™; electronic records in primary healthcare), and it includes sociodemographic characteristics, health conditions registered as International Classification of Diseases, 10th revision (ICD-10) codes, clinical parameters, toxic habits, laboratory data, and general practitioners’ prescriptions identified through anatomical therapeutic chemical (ATC) codes.

The information on the hospital environment corresponding to the centres located in Madrid will be obtained from different sources. In the case of Ramón y Cajal University Hospital, they will be obtained from the Conjunto Mínimo Básico de Datos (CMBD; Basic minimum set of data) and the laboratory data from the Openlab system. For Fundación Jiménez Díaz University Hospital, data will be obtained from the hospital information system HIS, which integrates clinical and laboratory data. Hospital Clínico San Carlos will get the clinical data from the system HIS Clinica and the laboratory data through the EoLIS System™.

For the Madrid hospitals, the data source for primary care will be obtained from the database that contains the information included in the Electronic Medical Record of Primary Care (AP-Madrid). This is a unique centralized electronic medical record containing clinical data from all primary care centers of Servicio Madrileño de Salud (SERMAS). It covers approximately 6.6 million people. It includes clinical data similar to that one from Catalonia except for diagnoses coding, which runs with the International Classification of Primary Care, 2nd revision (ICPC-2).

Detailed information about data sources is described in Table 1.

### Participants

Cases will be patients admitted to hospital with LA, that is defined by pH < 7.35 and plasmatic lactic acid concentration > 5 mM/L within the first 24 and 72 h after admission, respectively. These short observation periods are targeted to exclude LA that develops during hospitalization in patients admitted due to other reasons. The day of admission will be the index date. Inclusion criteria include: (1) 18-year-old or older, (2) hospital or primary healthcare diagnosis of DM2 previous to the index date (Additional file 1: Table S1), (3) moderate to severe CKD (stage 3a, 3b or 4 of the Kidney Disease Improving Global Outcomes [KDIGO] classification) [13] during the 2-year period before the index date (excluding the last 2 weeks; see below), taking into account data from the primary healthcare database, and (4) to have any information recorded on the primary healthcare database within the 1-year period prior to the index date. Patients will be excluded as cases if they have any of the following diagnosis: (1) diabetic ketoacidosis during the current in-hospital stay; (2) hospital or primary healthcare diagnosis of type 1 diabetes mellitus, human immunodeficiency virus disease or solid organ transplantation before the index date; (3) hospital or primary healthcare diagnosis of malignant neoplasm (except skin cancer other than melanoma; including pheochromocytoma) within the 5-year period prior to index date (Additional file 1: Table S1). In Catalonia, patients not registered in the hospital referral area will also be excluded.

Controls will be randomly selected from the population assigned to the primary health care area of the hospital cases and matched to them in a ratio 10:1 on age (within 2 years for cases aged 60 to 85-year-old, extendable to 3 years if necessary; cases older than 85 or younger than 60 years are matched to controls older than 85 or younger than 60, respectively), gender, CKD stage, and year (taking the date of admissions of the cases as a reference). The same index date of each case will be assigned to their controls. All the information needed to assess controls for eligibility criteria will be obtained from the primary healthcare databases. To be eligible, controls need to be 18-year-old or older, have DM2 diagnosis before the index date, and CKD stage as defined for cases during the 2-year period before the index date (excluding the last 2 weeks). Additionally, they need to have any information recorded on the primary healthcare database within the 2-year period prior to the index date. Exclusion criteria for controls include: (1) diagnosis of type 1 diabetes mellitus, human immunodeficiency virus disease or solid organ transplantation before the index date, (2) diagnosis of malignant neoplasm (except skin cancer other than melanoma; including pheochromocytoma) within the 5-year period prior to index date, and (3) patient not resident in the area of the study. A patient included in the study as a case will not be able to be included as a control.

### Variables

The following variables will be obtained from hospital databases:

- hospital admission data: admission date, discharge diagnoses, in-hospital death, admission to critical care unit;
- demographic data: age and sex;
- laboratory test data (values and dates): plasmatic lactic acid concentration (all values during the first 72 h from hospital admission), pH (all values during the first 24 h from hospital admission), haemoglobin (all values during the 30-day period before the index date and the first 24 h from admission).

The following variables will be obtained from primary healthcare databases:

- laboratory test data (values and dates): serum creatinine (all values between 2 years and 2 weeks before the index date), haemoglobin (all values during the 30-day period before the index date).
- prescriptions drug data during the 1-year period before the index date: prescriptions of metformin, other non-insulin antidiabetic drugs (NIADDs), insulin, diuretics, renin-angiotensin system (RAS) inhibitors, non-steroidal anti-inflammatory drugs (NSAIDs) and dates of prescription (initial and final) (Additional file 2: Table S2). The prescribed posology and the National Drug Code (NDC) are retrieved for metformin prescriptions; each NDC corresponds to specific strength and quantity of the drug.

Additionally, the following diagnoses and their corresponding dates will be obtained from both hospital and primary healthcare databases: DM2, type 1 diabetes mellitus, diabetic ketoacidosis, diabetic target organ damage, human immunodeficiency virus infection, organ transplantation, malignant neoplasm, alcohol use, acute alcohol intoxication, other intoxications (cyanide, methanol, ethilenglicol, diethilenglicol, propilenglicol), cocaine use, liver disease, acute myocardial infarction, heart failure, peripheral arterial disease, dyslipidemia, hypertension, cerebrovascular disease, dementia, hemiplegia, connective tissue disease, acute respiratory failure, chronic respiratory disease, chronic pulmonary obstructive disease, surgery, acute kidney failure, seizures, dehydration, diarrhoea, vomiting, gastroenteritis, gastroduodenal ulcer, shock, sepsis, thiamine deficit, and tests that require the use of iodine-based contrasts (Additional file 1: Table S1).

Charlson comorbidity index will be estimated from the average of the accumulated score based on the presence of determined comorbidities [14].

Renal function will be assessed taking into account values of serum creatinine concentration recorded in primary healthcare database obtained between 2 years and 2 weeks before the index date. The eGFR will be calculated using the CKD-EPI formula that takes into account the gender, the age, the plasmatic concentration of creatinine, and the race [15]. For this study, it will be assumed that no African American patients are included. A CKD stage was assigned to each GFR estimation, following the KDIGO classification (stage 1: eGFR ≥90 mL/min/1,73 m^2^; stage 2: eGFR 60–89 mL/min/1,73 m^2^; stage 3a: eGFR 45–59 mL/min/1,73 m^2^; stage 3b: eGFR 30–44 mL/min/1,73 m^2^; stage 4: eGFR 15–29 mL/min/1,73 m^2^; stage 5: < 15 mL/min/1,73 m^2^) [16]. In case of several eGFR estimates resulting in different CKD stages for an individual patient across the 2-year period, the CKD stage closer to the index date will be assigned to this patient.

### Exposure definition

Exposure to metformin, other NIADDs and insulin will be defined as prescriptions during the 365-day period prior to the index date and classified as current use (prescription during the 30-day period prior to the index date) or past use (prescription before the 30-days prior to the index date).

Starting and final prescription dates will define the length of the exposure. Consecutive prescriptions within 30 days will be considered the same exposure period. Consecutive prescriptions with a 30-day or longer gap will be considered as two different exposure periods.

The prescribed daily dose of metformin will be calculated taking into account the posology recorded by the prescriber and the strength of the drug prescribed pointed out by its National Drug Code. The calculated daily dose is categorized in < 1 g, 1-2 g, and > 2 g.

Other antidiabetic drugs will be classified into pharmacological subgroups.

Exposure to diuretics, RAS inhibitors, and NSAIDs will be defined as prescription during the 30-day period prior to the index date. Additional file 3 contains the STROBE checklist completed for this study protocol.

### Sample size calculation

Assuming a prevalence of metformin exposure of 40% in DM2 patients with moderate-severe CKD, it was calculated that 39 cases with 10 matched controls will be necessary to have a power of 90% for detecting a risk of LA associated with the use of metformin with an odds ratio ≥ 3 and a two-sided significance level of 0.05.

### Statistical analysis

Baseline characteristics will be described for cases and controls. For qualitative variables, absolute and relative frequencies will be provided. For quantitative variables, the main statistic parameters will be calculated (mean, standard deviation, median, interquartile range, minimum and maximum).

Unadjusted and adjusted risk of LA associated with metformin will be estimated through a stratified logistic regression. Odds ratio and 95% confidence intervals will be provided. The following covariables will be included in the model: age, gender, alcohol use, cocaine use, intoxications, severe anaemia, Charlson comorbidity index, complications of diabetes mellitus, liver disease, acute myocardial infarction, heart failure, surgery, anaesthesia, seizures, dehydration, vomiting, diarrhoea, gastroenteritis, sepsis, shock, thiamine deficit, acute respiratory failure, chronic obstructive pulmonary disease, acute renal failure, tests that require the use of iodine-based contrasts, exposure to oral hypoglycaemic agents other than metformin, to insulin, to diuretics, to RAS inhibitors, and to NSAIDs. Variable selection will be performed by a stepwise procedure.

Patients with complete data for these covariables will be taken into account for the main analysis. However, a sensitivity analysis will be performed using a Markov chain Monte Carlo method for multiple imputations to missing values.

Subgroup analyses will be performed according to daily dose (< 1 g, 1-2 g, and > 2 g), use levels (current and past use), disease stage (3a, 3b, and 4) and territory (Catalonia and Madrid).

As secondary analyses, the risk of LA associated with other hypoglycaemic drugs and insulin will also be estimated. Additionally, the overall case fatality rate of LA as well as the case fatality rate stratified by CKD stage will be calculated from the number of deaths among cases and the total number of cases.

The possibility of bias detection will be studied by analyzing the frequency of determination of plasmatic lactate levels in patients with metabolic acidosis according to the status of metformin exposure. This analysis will be performed with data from two of the participating hospitals in a sample of episodes of urgent hospital admission with pH < 7.35 during the first 24 h.

All statistical analyses will be performed with R statistical package version 3.4.0 or higher.

## Discussion

The ALIMAR-C2 study is a multicenter population-based case-control study using hospital and primary care health databases in order to assess the association between the use of metformin and LA in patients with DM2 and moderate to severe CKD.

Observational studies on the same exposure-outcome association using different databases could be inconsistent because of variations in methodological and record factors intrinsically related to the databases as well as differences in the health care system [17]. This study aims to analyze information collected from different electronic healthcare databases with administrative and clinical data retrieved from different assistance levels (hospitals and primary care) from two Spanish regions.

Study design and methodology are some of the factors contributing to the diversity and discrepancy of study results, even when using the same database [18]. When estimating the risk from different databases the way the information has been collected and then extracted could also make the results inconsistent. The homogeneous methodology that will be used when collecting data and the use of a uniform definition for drug exposure, outcome and confounders sharing the same protocol alongside the different data sets could bring more consistency to our results. All data will be analysed as if it came from one database.

On the other hand, when analyzing the exposures according to the prescriptions of medicines and not to the dispensation or to their actual intake, the exposure could be overestimated, but this would happen in the same way in cases and in controls [19]. Therefore, potential overestimation is not expected to have a net effect on the results.

Another important limitation, such as the existence of confounding factors, will be minimized by matching cases and controls for certain variables and performing a proper statistical analysis.

Regarding systematic errors that could bias a case-control study, the possible selection bias due to the difficulty of an adequate selection of controls is minimized since the controls are a random sample of the population where the cases come from, i.e. population with DM2 and moderate-severe CKD. Therefore, they are expected to have the same probability as the cases of having been exposed to metformin.

The possibility of observational bias should also be considered. If the diagnosis of LA or the determination of plasmatic lactate levels in a patient with acidosis is not actually performed symmetrically in patients exposed to metformin and in those not exposed, the prevalence of metformin exposure in cases of LA and therefore the strength of association could be overestimated [10]. This phenomenon is planned to be analyzed in our study as a secondary objective.

On the other hand, the major strengths of our study are the multicenter population-based design and our case definition, which is based on objective laboratory parameters at arrival to the hospital and unrelated to registered diagnoses.

In conclusion, LA has been considered an infrequent though serious adverse effect of metformin, with CKD pointed out as the most important risk factor. With this study, we try to provide additional evidence on the benefit-risk balance of metformin in DM2 patients with moderate to severe CKD.

## Additional files

Additional file 1: **Table S1.** Diagnosis codes according International Classification of Diseases version 9 (ICD-9) and 10 (ICD-10), and International Classification of Primary Care (ICPC-2) used to identify diagnoses. (DOCX 26 kb)

Additional file 2: **Table S2.** Anatomical Therapeutic Chemical Classification (ATC) codes of the drugs of interest. (DOCX 19 kb)

Additional file 3: STROBE Checklist. STROBE Checklist of Study Protocol. (DOCX 40 kb)

## Abbreviations

CKD: Chronic kidney disease
CMBD: Basic minimum set of data
CREC: Clinical Research Ethics Committee
DM2: Type 2 diabetes mellitus
eGFR: expected glomerular filtration rate
EMR: Electronic medical record
ENCePP: European Network of Centres for Pharmacoepidemiology and Pharmacovigilance
ER: Emergency Room
KDIGO: Kidney Disease Improving Global Outcomes
LA: Lactic acidosis
NIADD: Non-insulin antidiabetic drug
NSAID: Non-steroidal anti-inflammatory drug
PHC: Primary health care
RAS: Renin-angiotensin system
SAP BO: Systems, Applications and Products in Data Processing Business Objects

## References

[1] National Institute for Health and Clinical Excellence. Type 2 diabetes in adults: management. https://www.nice.org.uk/guidance/ng28/resources/type-2-diabetes-in-adults-management-pdf-1837338615493. Accessed 21 Nov 2018.

[2] UK Prospective Diabetes Study (UKPDS) Group (1998). Effect of intensive blood-glucose control with metformin on complications in overweight patients with type 2 diabetes (UKPDS 34). Lancet.

[3] Lalau J-D (2010). Lactic acidosis induced by metformin: incidence, management and prevention. Drug Saf.

[4] del Pozo-Fernández C, Pardo-Ruiz C, Sánchez-Botella C, Blanes-Castañer V, López-Menchero R, Gisbert-Sellés C (2012). Discrepancies among consensus documents, guidelines, clinical practice and the legal framework for the treatment of type 2 diabetes mellitus patients. Nefrologia.

[5] Bodmer M, Meier C, Krähenbühl S (2008). Metformin, sulfonylureas, or other antidiabetes drugs and the risk of lactic acidosis or hypoglicemia. Diabetes Care.

[6] Chang CH, Sakaguchi M, Dolin P (2016). Epidemiology of lactic acidosis in type 2 diabetes patients with metformin in Japan. Pharmacoepidemiol Drug Saf.

[7] Aharaz A, Pottegard A, Henriksen DP, Hallas J, Beck-Nielsen H, Lassen AT (2018). Risk of lactic acidosis in type 2 diabetes patients using metformin: a case control study. PLoS One.

[8] Richy FF, Sabidó-Espin M, Guedes S, Corvino FA, Gottwald-Hostalek U (2014). Incidence of lactic acidosis in patients with type 2 diabetes with and without renal impairment treated with metformin: a retrospective cohort study. Diabetes Care.

[9] Eppenga WL, Lalmohamed A, Geerts AF, Derijks HJ, Wensing M, Egberts A (2014). Risk of lactic acidosis or elevated lactate concentrations in metformin users with renal impairment: a population-based cohort study. Diabetes Care.

[10] Li L, Jick S, Gopalakrishnan C, Heide-Jørgensen U, Nørrelund H, Sørensen HT (2017). Metformin use and risk of lactic acidosis in people with diabetes with and without renal impairment: a cohort study in Denmark and the UK. Diabet Med.

[11] European Medicines Agency. Use of metformin to treat diabetes now expanded to patients with moderately reduced kidney function recommendations for patients with kidney impairment updated in product information. EMA/868987/2016. https://www.ema.europa.eu/documents/referral/metformin-article-31-referral-use-metformin-treat-diabetes-now-expanded-patients-moderately-reduced_en.pdf. Accessed 21 Nov 2018.

[12] Information System for Research in Primary Care. 2017. http://www.sidiap.org. Accessed 21 Nov 2018.

[13] Levey AS, Eckardt KU, Tsukamoto Y, Levin A, Coresh J, Rossert J, De Zeeuw D, Hostetter TH, Lameire N, Eknoyan G (2005). Definition and classification of chronic kidney disease: A position statement from Kidney Disease: Improving Global Outcomes (KDIGO). Kidney Int.

[14] Charlson ME, Pompei P, Ales KL, MacKenzie CR (1987). A new method of classifying prognostic comorbidity in longitudinal studies: development and validation. J Chronic Dis.

[15] Levey AS, Stevens LA, Schmid CH, Zhang YL, Castro AF, Feldman HI (2009). A new equation to estimate glomerular filtration rate. Ann Intern Med.

[16] Levin A, Stevens PE (2014). Summary of KDIGO 2012 CKD guideline: behind the scenes, need for guidance, and a framework for moving forward. Kidney Int.

[17] Souverein PC, Abbing-Karahagopian V, Martin E, Huerta C, de Abajo F, Leufkens HGM (2016). Understanding inconsistency in the results from observational pharmacoepidemiological studies: the case of antidepressant use and risk of hip/femur fractures. Pharmacoepidemiol Drug Saf.

[18] Abbing-Karahagopian V, Kurz X, de Vries F, van Staa TP, Alvarez Y, Hesse U (2014). Bridging differences in outcomes of pharmacoepidemiological studies: design and first results of the PROTECT project. Curr Clin Pharmacol.

[19] Schneeweiss S, Avorn J (2005). A review of uses of health care utilization databases for epidemiologic research on therapeutics. J Clin Epidemiol.

