# Supplementary material for: Lactic acidosis associated with metformin in patients with moderate to severe chronic kidney disease: study protocol for a multicenter population-based case-control study using health databases
Source: BMC Nephrol. 2019 May 30;20:193. doi: 10.1186/s12882-019-1389-8 (PMC6543584; doi:10.1186/s12882-019-1389-8)
Supplement: Supplementary file 1 — Table S1. Diagnosis codes according International Classification of Diseases version 9 (ICD-9) and 10 (ICD-10), and International Classification of Primary Care (ICPC-2) used to identify diagnoses. (DOCX 26 kb) [file 12882_2019_1389_MOESM1_ESM.docx]

**Table S1. Diagnosis codes according International Classification of Diseases version 9 (ICD-9) and 10 (ICD-10), and International Classification of Primary Care (ICPC-2) used to identify diagnoses**

| **Diagnosis** | **ICD-9** | **ICD-10** | **ICPC-2** |
| --- | --- | --- | --- |
| Acute alcohol intoxication | 303.0*  571.1*  980.0*  E860.0* to E860.1* | F10.0*  T51.0*  X45.*  X65.*  Y91.0* to Y91.3 | P16 |
| Acute myocardial infarction | 410.*  412.* | I21.* to I24.9*  I25.2* | K75 |
| Acute renal failure | 584.* | N17.* | - |
| Alcoholism | 303.9*  305.0*  571.0*  571.2* to 571.3* | F10.2* G31.2*  K70.*  Y91.9*  Z72.1* | P15 |
| Arterial hypertension | 362.11  401.* to 405.*  437.2* | I10.* to I15.* H35.0* | K85 to K87 |
| Cerebrovascular disease | 430.* to 438.* | G45.* to G46.*  I60.* to I67.1* | K90  K91 |
| Chronic obstructive pulmonary disease | 496.* | J43.* to J44.* | R95 |
| Chronic respiratory disease | 490.* to 496.*  500.* to 506.*  508.1*  515.* to 516.*  518.8* | J40.* to J47.* J60.* to J67.* J70.3*  M05.1* | R79  R96 |
| Cocaine use | 304.2*  305.6*  970.81 | F14.* | - |
| Connective tissue disease | 710.*  714.*  725.* | M05.* to M06.*  M30.* to M36.0* | L88 |
| Cyanide, ethylene glycol, diethylene glycol, propylene glycol, or methanol intoxication | 980.1*  982.8*  989.0*  E860.2*  E862.4*  E950.9* | T51.1*  T52.3*  T57.3*  T65.0* | - |
| Decompensated liver disease | 567.23  572.2*  789.5* | R18.* | - |
| Dehydration | 276.51 | E86.* | T11 |
| Dementia | 290.*  294.1*  331.0* to 331.2* | F00.* to F03.*  F05.1*  G30.*  G31.1* | P05  P70 |
| Diabetes mellitus type 1 | 250.01  250.03  250.21  250.23  250.31  250.33  250.41  250.43  250.51  250.53  250.61  250.63  250.71  250.73  250.81  250.83  250.91  250.93 | E10.* | T89 |
| Diabetes mellitus type 2 | 250.00  250.02  250.20  250.22  250.30  250.32  250.40  250.42  250.50  250.52  250.60  250.62  250.70  250.72  250.80  250.82  250.90  250.92 | E11.* | T90 |
| Diabetes with complications (and target organs complications) | 250.40  250.42  250.50  250.52  250.60  250.62  250.70  250.72  250.80  250.82  250.90  250.92  357.2*  362.01 to 362.07  366.41 | E11.0* to E11.8*  G59.0*  H28.0*  H36.0*  M14.2*  N08.3* | - |
| Diabetic ketoacidosis | 250.1* | - | - |
| Dyslipidemia | 272.0* to 272.4* | E78.0* to E78.5* | T93 |
| Gastroduodenal ulcer | 531.* to 534.* | K25.* to K28.* | D85  D86 |
| Gastroenteritis, vomiting or diarrhea | 003.0*  009.0* to 009.3*  558.*  564.3* to 564.5*  787.01  787.03 to 787.04  787.91 | A00.* to A09.* F50.5*  K52.*  K58.0*  K59.1*  K91.0.*  R11.* | D10  D11  D70  D73 |
| Generalized seizures | 345.1* to 345.5*  780.3* | G40.* to G41.*  R56.8 | N07 |
| Heart failure | 398.91  402.01  402.11  402.91  404.1*  404.3*  425.*  428.*  429.4* | I09.81 I11.0* I13.0*  I13.2  I42.*  I50.*  I97.13 | K77 |
| Hemiplegia | 342.*  438.2* | G81.* | N18 |
| Human immunodeficiency virus disease | 042.*  V08.*  795.71 | B20.* B21.* B22.* B23.* B24.*  R75.* Z21.* | B90 |
| Malignancy (excluding cutaneous malignacies other than melanoma; included pheochromocytoma) | 141.* to 172.*  174.* to 209.36  209.7*  230.*  233.* to 238.1*  238.3* to 239.1*  239.3* to 239.9*  258.01 to 258.03  511.81  789.51  V10.*  V71.1* | C01.* to C43.9* C45.* to C97.*  D00.* to D03.*  D05.* to D09.*  D37.* to D48.4^*^ D48.6* to D48.9* | A79  B72 to B74  D74 to D77 F74  H75 K72 L71 N74 N76 R84 R85 R92 T71 T73  U75 to U77 U79  W72  X75 to X77 X81  Y77 to Y78 |
| Mild liver disease | 070.1*  571.0* to 571.1*  571.3*  571.8* to 571.9*  573.1* to 573.2* | B15.9*  K70.0* to K70.2* K70.9*  K74.0* to K74.1* K76.0* to K76.1* K76.8* to K76.9* | D23 |
| Moderate-severe liver disease | 070.0*  070.2* to 070.7*  456.0* to 456.2*  567.23  570.*  571.2*  571.4* to 571.6*  572.0* to 573.*  789.5* | B15.0  B16.* to B19.0* I85.*  K70.3* to K70.4* K71.* to K73.* K74.2* to K75.* K76.3*  K76.6* to K76.7* R18.* | D97 |
| Organ transplantation | 00.91 to 00.93  07.94  11.60  11.69  33.5* to 33.6*  37.51  41.0*  41.94  46.97  50.5*  52.8*  55.6*  63.53  65.92  83.75  996.8*  V42.* V58.44  E878.0* | Z94.*  T86.*  Y83.0* | - |
| Peripheral arterial disease | 441.*  443.0* to 443.81  443.9* | I70.* I71.* I73.* I79.0*  Z95.8* to Z95.9* | K92 |
| Procedures with use of iodine contrast | 00.60 to 00.66  17.71  37.21 to 37.23  39.50  39.7*  39.90  44.44  87.02  87.05 to 87.08  87.13 to 87.15  87.21  87.31 to 87.35  87.38  87.5*  87.66  87.73 to 87.78  87.83 to 87.84  87.91  87.93 to 87.94  88.03 to 88.94  88.13 to 88.15  88.34  88.36  88.4* to 88.6* | - | - |
| Respiratory failure | 518.5*  518.81 to 518.82 | J80.*  J95.1* to J95.2* J96.0 | - |
| Sepsis | 038.*  785.52  995.91 to 995.92 | A40.* to A41.* | - |
| Shock | 785.5*  995.4*  995.6*  994.8*  639.5*  669.1*  998.00 to 998.02  998.09  994.0*  958.4* | A48.3* R57.* T78.0* T78.2* T88.2* T88.6* | - |
| Surgery and other procedures with general, spinal or epidural anesthesia | 00.3* to 00.48  00.50 to 00.58  00.60 to 00.66  00.7* to 00.9*  01.* to 16.*  17.1* to 17.6*  18.* to 20.*  21.04 to 21.1*  21.22 to 21.89  22.*  23.1*  25.2* to 25.9*  26.2* to 27.1*  27.3* to 28.0*  28.2* to 29.0*  29.2* to 33.4*  33.7* to 35.*  36.03  36.06 to 36.9*  37.1* to 37.4*  37.52 to 37.91  37.94 to 38.1*  38.3* to 38.8*  39.0* to 39.91  40.*  41.1* to 46.82  46.85 to 46.94  46.97 to 50.4*  51.* to 52.7*  52.9* to 54.95  55.0* to 55.5*  55.7* to 56.*  57.1* to 57.92  57.96 to 58.5*  58.9* to 62.7*  63.* to 67.0*  67.2* to 67.6*  68.0*  68.14 to 69.7*  69.93 to 70.1*  70.23 to 70.9*  71.5* to 71.9*  74.*  75.5* to 75.6*  75.91 to 75.93  76.* to 78.*  79.1* to 79.3*  79.5* to 79.6*  79.8* to 83.95  83.99 to 85.0*  85.2* to 85.9*  86.4* to 86.99 | - | - |
| Thiamine deficiency | 265.* | E51.* | - |
