# Supplementary material for: Lactic acidosis associated with metformin in patients with moderate to severe chronic kidney disease: study protocol for a multicenter population-based case-control study using health databases
Source: BMC Nephrol. 2019 May 30;20:193. doi: 10.1186/s12882-019-1389-8 (PMC6543584; doi:10.1186/s12882-019-1389-8)
Supplement: Supplementary file 2 — Table S2. Anatomical Therapeutic Chemical Classification (ATC) codes of the drugs of interest. (DOCX 19 kb) [file 12882_2019_1389_MOESM2_ESM.docx]

**Table S2. Anatomical Therapeutic Chemical Classification (ATC) codes of the drugs of interest**

| **Code ATC** | **Pharmacological subgroups** | **Drugs** |
| --- | --- | --- |
| A10BA02 | - | Metformin |
| A10BB | Sulfonylureas | - |
| A10BD02 | - | Metformin and sulfonylureas |
| A10BD03 | - | Metformin and rosiglitazone |
| A10BD04 | - | Glimepiride and rosiglitazone |
| A10BD05 | - | Metformin and pioglitazone |
| A10BD06 | - | Glimepiride and pioglitazone |
| A10BD07 | - | Metformin and sitagliptin |
| A10BD08 | - | Metformin and vildagliptin |
| A10BD09 | - | Pioglitazone and alogliptin |
| A10BD10 | - | Metformin and saxagliptin |
| A10BD11 | - | Metformin and linagliptin |
| A10BD13 | - | Metformin and alogliptin |
| A10BD14 | - | Metformin and repaglinide |
| A10BD15 | - | Metformin and dapagliflozin |
| A10BD16 | - | Metformin and canagliflozin |
| A10BD17 | - | Metformin and acarbose |
| A10BD18 | - | Metformin and gemigliptin |
| A10BD20 | - | Metformin and empagliflozin |
| A10BF | Alpha glucosidase inhibitors | - |
| A10BG | Thiazolidinediones | - |
| A10BH | Dipeptidyl peptidase 4 inhibitors | - |
| A10BX | Other blood glucose lowering drugs, excluding insulins | - |
| A10X | Other drugs used in diabetes | - |
| A10A | [Insulins and analogues](https://www.whocc.no/atc_ddd_index/?code=A10A) | - |
| M01A | Antiinflammatory and antirheumatic products, non-steroids | - |
| N02BA01 | - | Acetylsalicylic acid |
| C03 | Diuretics | - |
| C09 | Agents acting on the renin-angiotensin system | - |
