# Supplementary material for: Lactic acidosis associated with metformin in patients with moderate to severe chronic kidney disease: study protocol for a multicenter population-based case-control study using health databases
Source: BMC Nephrol. 2019 May 30;20:193. doi: 10.1186/s12882-019-1389-8 (PMC6543584; doi:10.1186/s12882-019-1389-8)
Supplement: Supplementary file 3 — STROBE Checklist. STROBE Checklist of Study Protocol. (DOCX 40 kb) [file 12882_2019_1389_MOESM3_ESM.docx]

STROBE Statement—Checklist of items that should be included in reports of ***case-control studies***

|  | Item No | Recommendation | Section, paragraph, page |
| --- | --- | --- | --- |
| **Title and abstract** | 1 | (*a*) Indicate the study’s design with a commonly used term in the title or the abstract | Title, page 1 |
|  |  | (*b*) Provide in the abstract an informative and balanced summary of what was done and what was found | Abstract, page 3 |
| Introduction | | |  |
| Background/rationale | 2 | Explain the scientific background and rationale for the investigation being reported | Background, paragraphs 1-5, pages 4-5 |
| Objectives | 3 | State specific objectives, including any prespecified hypotheses | Background, paragraph 6, page 5  Methods/design, Aims, page 6 |
| Methods | | |  |
| Study design | 4 | Present key elements of study design early in the paper | Methods/design, study design, paragraph 1, page 6 |
| Setting | 5 | Describe the setting, locations, and relevant dates, including periods of recruitment, exposure, follow-up, and data collection | Methods/design, setting, pages 6-7  Table 1 |
| Participants | 6 | (*a*) Give the eligibility criteria, and the sources and methods of case ascertainment and control selection. Give the rationale for the choice of cases and controls | Methods/design, participants, pages 8-10 |
|  |  | (*b*) For matched studies, give matching criteria and the number of controls per case | Methods/design, participants, paragraph 3, page 9. |
| Variables | 7 | Clearly define all outcomes, exposures, predictors, potential confounders, and effect modifiers. Give diagnostic criteria, if applicable | Methods/design, variables, pages 10-12  Methods/design, exposure definition, page 12  Methods/design, statistical analysis, paragraphs 2,4, pages 13-14 |
| Data sources/ measurement | 8* | For each variable of interest, give sources of data and details of methods of assessment (measurement). Describe comparability of assessment methods if there is more than one group | Methods/design, Data sources, pages 7-8; Table 1, additional file 1 “Table 1_LAinCKD_PedrosC.docx”   - Methods/design, Variables, pages 10-12; Table S1, additional file 2 “Supplementary Material Table1_LAinCKD_PedrosC.docx”; Table S2, additional file 3 “Supplementary MaterialTable2__LAinCKD_PedrosC.docx” - Methods/design, Exposure definition, page 12 |
| Bias | 9 | Describe any efforts to address potential sources of bias | Methods/design, statistical analysis, paragraph 6, page 14 |
| Study size | 10 | Explain how the study size was arrived at | Methods/design, statistical analysis, sample size calculation, page 13 |
| Quantitative variables | 11 | Explain how quantitative variables were handled in the analyses. If applicable, describe which groupings were chosen and why | Methods/design, statistical analysis, paragraphs 1-5, pages 13-14 |
| Statistical methods | 12 | (*a*) Describe all statistical methods, including those used to control for confounding | Methods/design, statistical analysis, pages 13-14 |
|  |  | (*b*) Describe any methods used to examine subgroups and interactions | Methods/design, statistical analysis, paragraphs 2,4, pages 13-14 |
|  |  | (*c*) Explain how missing data were addressed | Methods/design, statistical analysis, paragraph 3, page 14 |
|  |  | (*d*) If applicable, explain how matching of cases and controls was addressed | Methods/design, participants, paragraph 3, page 9. |
|  |  | (*e*) Describe any sensitivity analyses | Methods/design, statistical analysis, paragraph 3, page 14 |
| Results | | |  |
| Participants | 13* | (a) Report numbers of individuals at each stage of study—eg numbers potentially eligible, examined for eligibility, confirmed eligible, included in the study, completing follow-up, and analysed | NA |
|  |  | (b) Give reasons for non-participation at each stage | NA |
|  |  | (c) Consider use of a flow diagram | NA |
| Descriptive data | 14* | (a) Give characteristics of study participants (eg demographic, clinical, social) and information on exposures and potential confounders | NA |
|  |  | (b) Indicate number of participants with missing data for each variable of interest | NA |
| Outcome data | 15* | Report numbers in each exposure category, or summary measures of exposure | NA |
| Main results | 16 | (*a*) Give unadjusted estimates and, if applicable, confounder-adjusted estimates and their precision (eg, 95% confidence interval). Make clear which confounders were adjusted for and why they were included | NA |
|  |  | (*b*) Report category boundaries when continuous variables were categorized | NA |
|  |  | (*c*) If relevant, consider translating estimates of relative risk into absolute risk for a meaningful time period | NA |
| Other analyses | 17 | Report other analyses done—eg analyses of subgroups and interactions, and sensitivity analyses | NA |
| **Discussion** | | | |
| Key results | 18 | Summarise key results with reference to study objectives | NA |
| Limitations | 19 | Discuss limitations of the study, taking into account sources of potential bias or imprecision. Discuss both direction and magnitude of any potential bias | Discussion, paragraphs 2-6, pages 15-16. |
| Interpretation | 20 | Give a cautious overall interpretation of results considering objectives, limitations, multiplicity of analyses, results from similar studies, and other relevant evidence | NA |
| Generalisability | 21 | Discuss the generalisability (external validity) of the study results | NA |
| Other information | | |  |
| Funding | 22 | Give the source of funding and the role of the funders for the present study and, if applicable, for the original study on which the present article is based | Declarations, Funding , page 19 |

*Give information separately for cases and controls.
